# Subclinical dose irradiation triggers human breast cancer migration via mitochondrial reactive oxygen species

**DOI:** 10.1186/s40170-024-00347-1

**Published:** 2024-07-08

**Authors:** Justin D. Rondeau, Justine A. Van de Velde, Yasmine Bouidida, Pierre Sonveaux

**Affiliations:** 1 Pole of Pharmacology and Therapeutics, Institut de Recherche Expérimentale et Clinique (IREC), Université catholique de Louvain (UCLouvain), Brussels, 1200 Belgium; 2 WELBIO Department, WEL Research Institute, Wavre, 1300 Belgium

**Keywords:** Subclinical dose irradiation, Breast cancer, Mitochondria, Reactive oxygen species (ROS), Migration, MitoQ, Catalase

## Abstract

**Background:**

Despite technological advances in radiotherapy, cancer cells at the tumor margin and in diffusive infiltrates can receive subcytotoxic doses of photons. Even if only a minority of cancer cells are concerned, phenotypic consequences could be important considering that mitochondrial DNA (mtDNA) is a primary target of radiation and that damage to mtDNA can persist. In turn, mitochondrial dysfunction associated with enhanced mitochondrial ROS (mtROS) production could promote cancer cell migration out of the irradiation field in a natural attempt to escape therapy. In this study, using MCF7 and MDA-MB-231 human breast cancer cells as models, we aimed to elucidate the molecular mechanisms supporting a mitochondrial contribution to cancer cell migration induced by subclinical doses of irradiation (< 2 Gy).

**Methods:**

Mitochondrial dysfunction was tested using mtDNA multiplex PCR, oximetry, and ROS-sensitive fluorescent reporters. Migration was tested in transwells 48 h after irradiation in the presence or absence of inhibitors targeting specific ROS or downstream effectors. Among tested inhibitors, we designed a mitochondria-targeted version of human catalase (mtCAT) to selectively inactivate mitochondrial H_2_O_2_.

**Results:**

Photon irradiation at subclinical doses (0.5 Gy for MCF7 and 0.125 Gy for MDA-MB-231 cells) sequentially affected mtDNA levels and/or integrity, increased mtROS production, increased *MAP2K1/MEK1* gene expression, activated ROS-sensitive transcription factors NF-κB and AP1 and stimulated breast cancer cell migration. Targeting mtROS pharmacologically by MitoQ or genetically by mtCAT expression mitigated migration induced by a subclinical dose of irradiation.

**Conclusion:**

Subclinical doses of photon irradiation promote human breast cancer migration, which can be countered by selectively targeting mtROS.

**Supplementary Information:**

The online version contains supplementary material available at 10.1186/s40170-024-00347-1.

## Introduction

X-ray radiotherapy with or without (neo)adjuvant hormonal therapy and/or chemotherapy is a gold standard treatment option for women with breast cancer. However, treatment efficacy is limited by intrinsic and acquired radioresistance, an escape mechanism embroiled in intensive research [1–6]. A less studied escape mechanism is the potential for radiotherapy to stimulate cancer cell migration based on a natural tentative of cancer cells to leave the irradiation field. Modern advances in intensity-modulated radiotherapy [7] limit this possibility, as precise and accurate photon dose deposition can be achieved for most cancer cells within primary breast tumors. However, dose deposition is less accurate at the tumor margin and for cancer cells in diffusive infiltrates [8]. This is inherent to limited imaging resolution especially under breathing movements, making it difficult to precisely localize and target the tumor margin [9–11]. Peripheral cancer cells may thus receive subcytotoxic doses of photons, adapt, and escape.

To acquire migratory capacities is a first and key step towards exiting the irradiation field. While doses used for fractionated radiotherapy (1.8 to 2 Gy for conventional fractionation, 1.5 Gy bid for hypofractionation in inflammatory breast cancer, and ≥ 2.1 Gy for postoperative hypofractionation [12, 13]) are generally (but not always) detrimental to migration for radiosensitive cancer cells [14, 15], lower subcytotoxic doses can induce breast cancer cell proliferation [16], migration and invasion [17, 18]. With respect to breast cancer cell migration, the main mechanism identified to date is induction of an epithelial-to-mesenchymal transition [17, 18], with N-cadherin, vimentin, focal adhesion kinase signaling and nuclear β-catenin contributing to the migratory phenotype [19].

In the present study, we tested the hypothesis that mitochondria within breast cancer cells are a promigratory signaling hub activated by subclinical doses of ionizing radiation (defined here as < 2 Gy) based on two paradigms. The first is the increased vulnerability of mitochondrial DNA (mtDNA) to irradiation, due to the fact that mtDNA is principally composed of coding regions, is not protected by histones, and has limited repair capabilities compared to nuclear DNA [20]. Irradiation-induced mitochondrial dysfunction could thus persist and propagate until full mitochondrial turnover (fission, mitophagy, mitochondrial biogenesis and fusion) [21]. The second paradigm is that an increased subcytotoxic production of mitochondrial reactive oxygen species (mtROS) is sufficient to trigger breast cancer cell migration [22, 23]. mtROS mainly originate from the mitochondrial electron transport chain (ETC), and either an increased or a decreased ETC activity following bottlenecking damage results in enhanced electron leak [22]. Leaking electrons create mtROS, which collectively promote cancer cell migration by activating redox-sensitive effectors, including the transforming growth factor β (TGFβ) pathway [22]. Mitochondrial dysfunction can thus support sustained cancer cell migration, but whether subclinical doses of radiotherapy facilitate this event in breast cancer cells is currently unknown. We explored and validated this possibility in vitro using two different types of human breast cancer cells (luminal A and triple-negative). We report that mtROS can be genetically and pharmacologically targeted to block the gain in migration induced by subclinical doses of radiation.

## Methods

### Cells and cell culture

MCF7 (catalog #HTB-22) and MDA-MB-231 (catalog #HTB-26) human breast cancer cells from the American Type Culture Collection were routinely cultured in DMEM-GlutaMAX medium (Gibco, catalog #61965-026) containing 4.5 g/L *D*-glucose without pyruvate, supplemented with 10% FBS. Cultures were maintained at 37 °C in a 5% CO_2_ humidity-controlled incubator, with regular checks to verify the absence of *mycoplasma* (MycoAlert Plus, Lonza; catalog #LT07-710). Cell counting was performed on a Spectramax i3x spectrophotometer equipped with a MiniMax imaging cytometer (Molecular Devices).

### Drugs

Mitoquinone mesylate (MitoQ) was a kind gift of Michael P. Murphy (University of Cambridge, UK). IκB kinase (IKK) inhibitor BMS-345541 (MedChemExpress, catalog #HY-10,519) was used to repress nuclear factor-κB (NF-κB) activity, and c-Jun inhibitor T-52241 (MedChemExpress, catalog #HY-12,270) to repress activating protein 1 (AP1) activity. Unless stated otherwise, all other drugs were from Sigma-Aldrich.

### Vectors and transfection

Cells were transfected 1 h before irradiation using lipofectamine 3000 (Thermo Fisher, catalogue #L3000001) according to manufacturer’s protocol. For experiments targeting mitochondrial H_2_O_2_ (mtH_2_O_2_), we constructed a vector encoding a mitochondria-targeted version of human HA-tagged catalase (mtCAT) following the procedures detailed in Supplementary Methods. Control empty vector was pCMV3-C-HA (Sino Biological, catalog #CV013). Vectors reporting on the transcriptional activities of NF-κB and AP1/c-Jun were 4xNFkB Luc (Addgene, catalog #111,216) and 3xAP1pGL3 (Addgene, catalog #40,342), respectively. Negative control was pGL4-23-NegCtrl (Addgene, catalog #163,904). pTK-Green Renilla (Thermo Fisher Scientific, catalog #16,154) was used to normalize for transfection efficiency. Assays were performed 24 h after transfection.

### Irradiation

Adherent cells in culture dishes were irradiated at a dose rate of 0.8 Gy/min using an IBL-637 ^137^Cs photon irradiator (Gamma Service Medica). They were allowed to recover for 24 h before any other experimental intervention.

### Migration and invasion

Migration and invasion were assayed in 24-well transwell plates with 8.0 μm pore size inserts (Corning, catalog #353,097) with 0.2% (MDA-MB-231) or 10% (MCF7) FBS as chemoattractant, as previously reported [24]. After 24 h of migration or invasion in the presence of tested pharmacological agents, cells at the bottom of the insert were fixed with 4% paraformaldehyde (PFA) for 10 min, washed twice with PBS, and stained with 0.5% crystal violet for 2 h. Remaining cells at the top of the insert were removed with a cotton swab. Pictures were taken at 5x magnification on a Zeiss Axiovert S100 microscope and quantified using QuPath version 0.2.3 (University of Edinburgh). All results are expressed as % of the basal migration of untreated cells.

### mtDNA quantification

The quantification of total and deleted mtDNA (common 4977 bp deletion) was performed using multiplex PCR on a ViiA 7 Real-Time PCR system (Applied Biosystems), using a previously described protocol [25]. Briefly, primers encoding sequences from the minor arc (total mtDNA) and major arc (damaged mtDNA) of the mitochondrial genome were amplified and quantified with FAM and NED fluorescent probes, respectively. Data were normalized to nuclear DNA levels (nuclear gene *β2M* detected with the VIC probe).

### Oximetry

Cellular oxygen consumption rates (OCRs) were determined using a Seahorse XFe96 bioenergetic analyzer (Agilent Technologies), according to manufacturer’s protocol. Briefly, 24 h after irradiation or sham, 10,000 MCF7 or 5,000 MDA-MB-231 cells were seeded in their routine culture medium in XFe96 culture plates, treated pharmacologically as indicated, and left to adhere for 24 h. Cells where then assayed in CO_2_-free DMEM containing 10 mM glucose, 2 mM glutamine, 1.85 g/L NaCl, 3 mg/L phenol red, pH 7.4, using the XF cell MitoStress kit (Agilent Technologies) in the presence of indicated pharmacological modulators. Mitochondrial OCR (mtOCR) was calculated as the difference between basal OCR and nonmitochondrial OCR measured upon full ETC inhibition by 0.5 µM of Complex I inhibitor rotenone + 0.5 µM of Complex III inhibitor antimycin A.

### Glucose and lactate measurements

Glucose uptake and lactate secretion rates were determined by measuring glucose and lactate concentrations in cell medium 48 h after treatment using a CMA600 enzymatic analyzer (Aurora Borealis), as previously described [26].

### ROS measurements

Twenty-four hours after irradiation 2 × 10^5^ MCF7 or 10^5^ MDA-MB-231 cells were seeded in complete medium and allowed to adhere in black, clear-bottom 96-well plates (Greiner Bio One). Whole cell ROS levels were determined using dihydroethidium (DHE; Abcam, catalog #ab236206), mtROS using MitoSOX (Thermo Fisher Scientific, catalog #M36008) [27], and mitochondrial H_2_O_2_ using mitochondria peroxy yellow 1 (MitoPY1; Biotechne, catalog #4428) [28]. Fluorescence was measured on a Spectramax i3x spectrophotometer equipped with a MiniMax imaging cytometer. All data are expressed as % of unirradiated controls.

### Generation of mitochondrial H_2_O_2_

To selectively generate H_2_O_2_ in mitochondria, cells were transfected with a mtHyPer-*D*-amino acid oxidase (DAAO) plasmid (Addgene, catalog #168,304) [29] using lipofectamine 3000. Within mitochondria, flavoenzyme DAAO generates H_2_O_2_ by catalyzing the conversion of exogenously supplied *D*-alanine, but not *L*-alanine, to pyruvate, and the fluorescent sensor HyPer selectively reports on mtH_2_O_2_ levels [30], which were measured on a Spectramax i3x spectrophotometer equipped with a MiniMax imaging cytometer.

### Real-time quantitative PCR

Total mRNA was extracted and quantified as previously reported [23]. Primers were for *MAP2K1* forward 5’-GGG-ACC-AGC-TCT-GCG-GAG-A-3’; reward 5’-GCC-CCC-AGC-TCA-CTG-ATC-TTC-T-3’, and for *HPRT* forward 5’-TGG-CGT-CGT-GA-TAG-TGA-TG-3’ and reward R: 5’-CAC-CCT-TTC-CAA-ATC-CTC-AG-3’). All data were normalized to *HPRT* gene expression.

### Apoptosis detection

Apoptosis was detected using previously disclosed protocols that are detailed in the Supplementary Methods.

### Statistical analyses

All results are expressed as means ± standard error of the mean (SEM) for *n* independent observations. Error bars are sometimes smaller than symbols. Outliers were identified using Dixon’s Q test. Data were analyzed using GraphPad Prism 8.4.3. Student’s *t* test and one-way ANOVA were used where appropriate. *P* < 0.05 was considered to be statistically significant.

## Results

### Irradiation at subclinical doses promotes human breast cancer cell migration and mtROS production

**Fig. 1:**
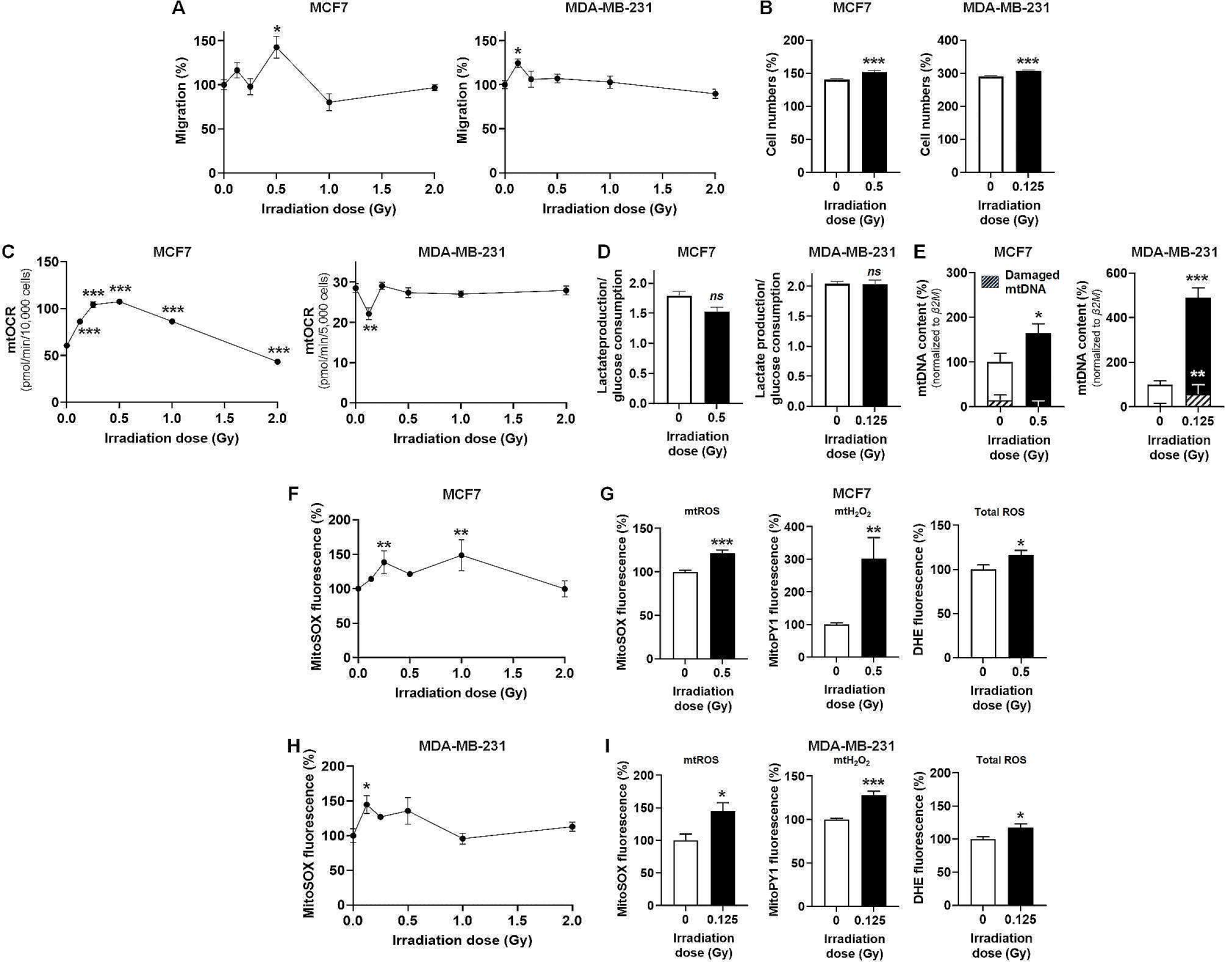
Subclinical doses of irradiation stimulate human breast cancer cell migration, alter respiration and trigger mtROS production. (**A**) Cancer cells were irradiated with increasing doses of photons. Their migratory capacities were assayed in transwells for a duration of 24 h starting 24 h after irradiation, with FBS as chemoattractant. MCF7 migration is shown on the left (*n* = 4) and MDA-MB-231 on the right graph (*n* = 8). (**B**) MCF7 (left graph, *n* = 10) and MDA-MB-231 (right graph, *n* = 10) cells were counted 48 h after irradiation with a single dose of 0.5 Gy and 0.125 Gy, respectively. (**C**) The mitochondrial oxygen consumption rate (mtOCR) of 10,000 MCF7 cell (left graph, *n* = 5) and 5,000 MDA-MBA-231 cells (right graph, *n* = 5) was measured on a Seahorse bioenergetic analyzer 48 h after increasing doses of irradiation. (**D**) Glucose consumption and lactate release rates were determined enzymatically 48 h after irradiation. The left graph shows the lactate production/glucose consumption ratio for MCF7 (*n* = 3) and the right graph for MDA-MB-231 (*n* = 3) cells. (**E**) Multiplex PCR was used to quantify total mtDNA and damaged mtDNA (common deletion) levels in MCF7 (*n* = 6) and MDA-MB-231 (*n* = 6) cells 48 h after irradiation. (**F**) In MCF7 cells, mitochondrial ROS (mtROS) levels were measured using MitoSOX fluorescence 48 h after increasing doses of irradiation (*n* = 3–10). (**G**) In MCF7 cells, mtROS levels were measured using MitoSOX fluorescence 48 h after irradiation (left graph, *n* = 10), mitochondrial H_2_O_2_ (mtH_2_O_2_) levels using MitoPY1 fluorescence (middle graph, *n* = 8), and whole cell ROS levels using dihydroethidium (DHE) fluorescence (right graph, *n* = 18). (**H**) As in (F) but using MDA-MB-231 cells (*n* = 8). (**I**) As in (G) but using MDA-MB-231 cells (left graph, *n* = 8; middle graph, *n* = 9; right graph, *n* = 18). All data are shown as means ± SEM. * *P* < 0.05, ** *P* < 0.01, *** *P* < 0.005, *ns P* > 0.05; by one-way ANOVA with Dunnett post-hoc test (A, C, F, H) or by Student’s *t* test (B, D, E, G, I)

To test whether subclinical doses of irradiation could promote cancer cell migration, luminal A MCF7 and triple-negative MDA-MB-231 human breast cancer cells were irradiated at photon doses ranging from 0.125 to 2 Gy (2 Gy being a reference clinical dose [12]) and assayed in transwells with FBS as chemoattractant. Peaks in migration were detected 48 h after 0.5 Gy for MCF7 (+ 42.6 ± 12.3%) and 48 h after 0.125 Gy for MDA-MB-231 (+ 24.5 ± 13.7%) cells (Fig. 1a). These doses were subcytotoxic (Fig. 1b). They did not induce breast cancer cell invasion in transwells (Figure S1). Metabolically, photon irradiation dose-dependently increased the mtOCR of MCF7 cells, which peaked 48 h after 0.5 Gy (Fig. 1c). Conversely, MDA-MB-231 cell mtOCR was significantly decreased 48 h after 0.125 Gy. The glycolytic rate (lactate/glucose ratio) of the two cell lines was unchanged (Fig. 1d). The increased mtOCR of MCF7 cells could be explained by an increased mitochondrial quality (more undamaged mtDNA), whereas the decreased mtOCR of MDA-MB-231 cells was associated with persistent mtDNA damage (common deletion) despite an increased total mtDNA content (Fig. 1e).

With respect to irradiation doses, maximal effects on mtOCR (Fig. 1c) correlated with maximal effects on migration (Fig. 1a). In the same conditions, mtROS levels were induced in MCF7 cells for doses ranging from 0.25 to 1 Gy (Fig. 1f). Actually, mtROS, mtH_2_O_2_ and total ROS levels were all significantly increased 48 h after 0.5 Gy (Fig. 1g). In MDA-MB-231 cells, the migration and mtOCR peaks observed at 0.125 Gy closely corresponded to the maximal mtROS levels also observed at 0.125 Gy (Fig. 1h). mtROS, mtH_2_O_2_ and total ROS levels were all significantly increased 48 h after 0.125 Gy (Fig. 1i). This indicated that mtROS could participate in the migratory response of breast cancer cells irradiated at subclinical doses.

### Targeting mtROS inhibits irradiation-induced breast cancer cell migration

**Fig. 2:**
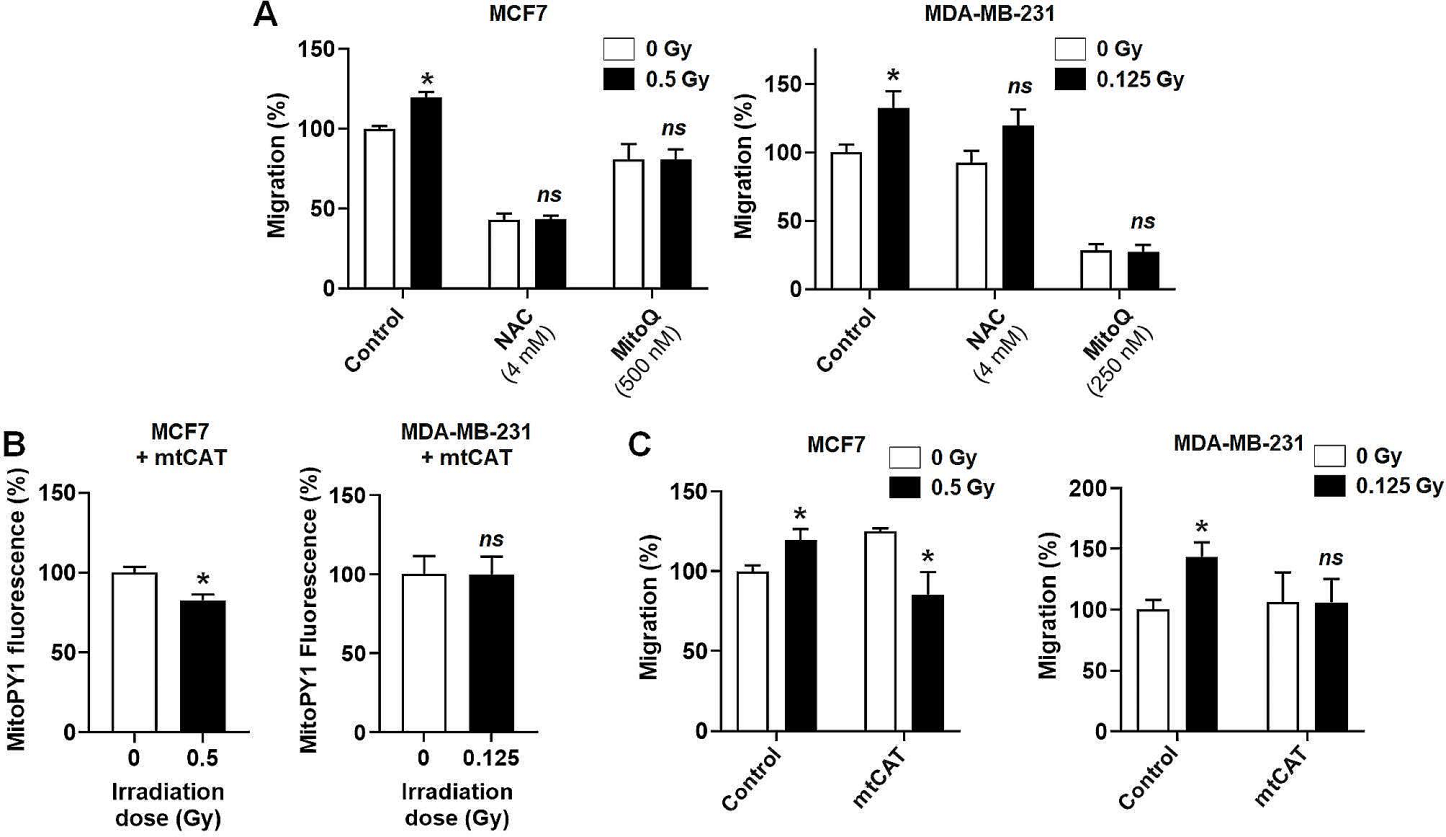
Targeting ROS inhibits the human breast cancer cell migration induced by subclinical doses of irradiation. (**A-C**) MCF7 and MDA-MB-231 were irradiated or not with a single dose of 0.5 Gy and 0.125 Gy, respectively. (**A**) Where indicated, cells were treated with general antioxidant *N*-acetyl-*L*-cysteine (NAC, 4 mM) or mtROS inhibitor MitoQ (500 nM for MCF7 and 250 nM for MDA-MB231 cells) starting 24 h after irradiation and during a 24 h migration in transwells with FBS as chemoattractant. MCF7 cell migration is shown on the left (*n* = 4) and MDA-MB cell migration on the right graph (*n* = 7–8). (**B**) Twenty-four hours after irradiation, MCF7 and MDA-MB-231 cells were transfected with a plasmid encoding a mitochondria-targeted version of catalase (mtCAT). Mitochondrial H_2_O_2_ measured 24 h later using MitoPY1 fluorescence is shown in the left graph for MCF7 (*n* = 4) and in the right graph for MDA-MB-231 (*n* = 5) cells. (**C**) Cells were transfected or not with a plasmid encoding mtCAT 1 h after irradiation, left to recover for 24 h, and then assayed for migration for 24 h in transwells with FBS as chemoattractant. Migration is shown in the left graph for MCF7 (*n* = 3–4) and in the right graph for MDA-MB-231 (*n* = 6) cells. All data are shown as mean ± SEM. * *P* < 0.05, *ns P* > 0.05, by Student’s *t* test (**A**-**C**)

Whether migration induced by subclinical doses of irradiation depends on mtROS production was tested using *N*-acetyl-*L*-cysteine (NAC, a general antioxidant) and MitoQ (selectively targeting mtROS) [31]. The two antioxidants inhibited basal and irradiation-induced breast cancer cell migration, with NAC being more effective for MCF7 and MitoQ for MDA-MB-231 cells (Fig. 2a). In general, mitochondrial superoxide has a very short half-life, as it is rapidly converted to H_2_O_2_ by mitochondrial superoxide dismutase 2 (SOD2) [32]. Whether mtH_2_O_2_ is involved in the breast cancer cell migration induced by subclinical doses of irradiation was tested using a mitochondria-targeted version of human catalase (mtCAT) (Figure S2 and Supplementary Methods), which effectively blocked irradiation-induced mtH_2_O_2_ production by both cell lines (Fig. 2b). Downstream, mtCAT completely inhibited irradiation-induced MCF7 and MDA-MB-231 cancer cell migration (Fig. 2c). Collectively, we concluded at this stage that subclinical doses of radiation trigger human breast cancer cell migration by inducing long-lasting ETC dysfunction, resulting in enhanced mitochondrial superoxide and mtH_2_O_2_ production.

### Generating H_2_O_2_ within mitochondria stimulates breast cancer cell migration

**Fig. 3:**
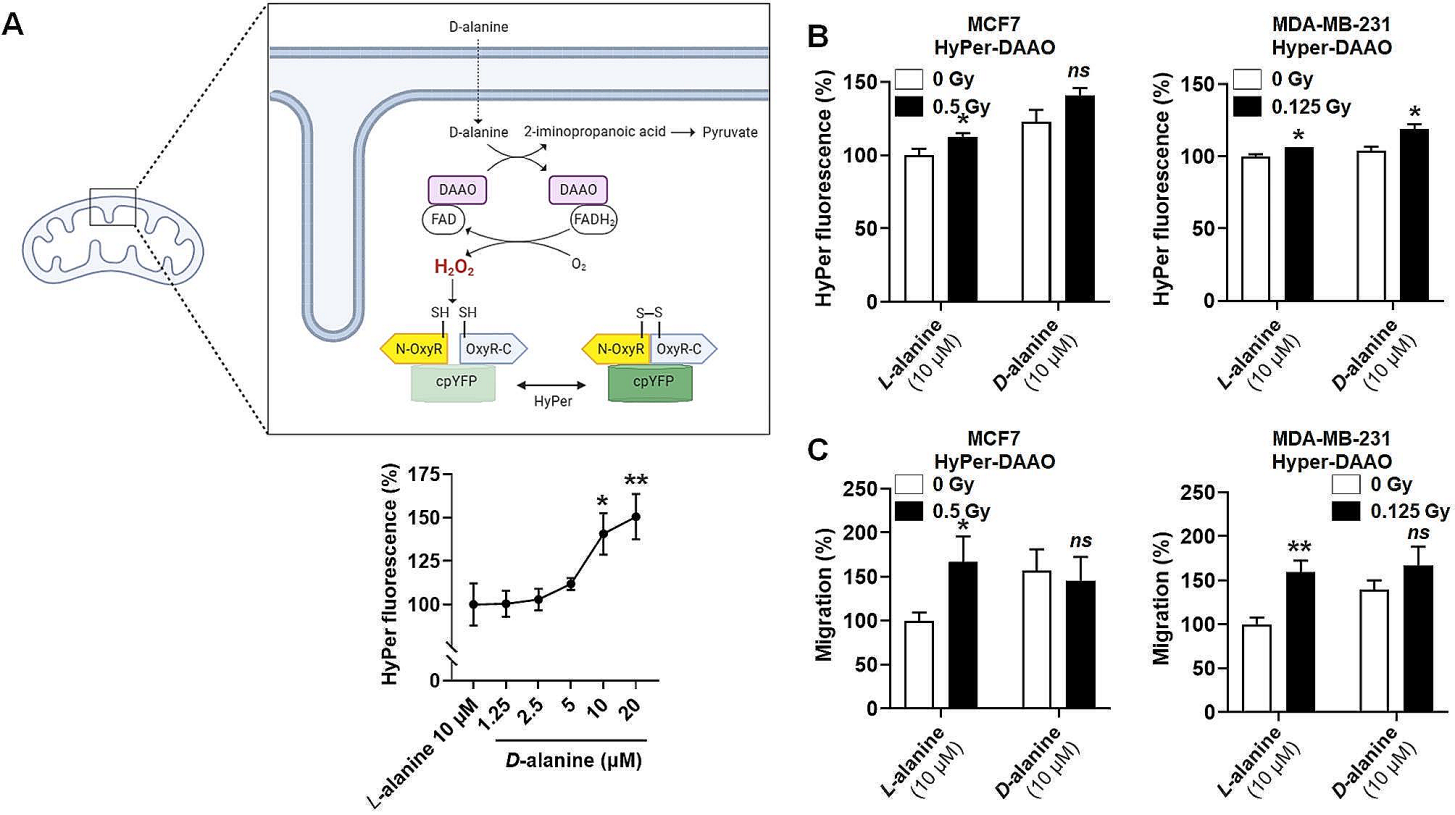
Enhancing H_2_O_2_ generation in mitochondria triggers human breast cancer cell migration. (**A**) Cartoon (produced using BioRender) depicting the mtDAAO-HyPer system used to generate (DAAO reaction fueled by exogenous *D*-alanine) and detect (HyPer fluorescent reporter) H_2_O_2_ selectively in cell mitochondria, based on previously reported data [29]. The graph shows a standard curve (F_500_/F_420_ HyPer fluorescence) generated 1 h after providing increasing doses of *D*-alanine to MCF7 cells expressing the mtDAAO-HyPer system, with 10 mM of *L*-alanine serving as a negative control (*n* = 5). (**B-C**) MCF7 and MDA-MB-231 were irradiated or not with a single dose of 0.5 Gy and 0.125 Gy, respectively, transfected with the mtDAAO-HyPer system 1 h later, and left to recover for 24 h before treatment with *L-*alanine (10 mM) or *D*-alanine (10 mM). (**B**) One hour later, HyPer fluorescence was measured in MCF7 (left graph, *n* = 5) and MDA-MB-231 (right graph, *n* = 5) cells. (**C**) After irradiation and transfection, MCF7 (left graph, *n* = 12) and MDA-MB-231 (right graph, *n* = 8–9) cell migration was determined over 24 h in transwells with FBS as chemoattractant in the presence of either *L*-alanine or *D*-alanine. All data are shown as means ± SEM. * *P* < 0.05, ** *P* < 0.01 compared to control; by one-way ANOVA with Dunnett post-hoc test (**A**) or by Student’s *t* test (**B**, **C**)

A corollary hypothesis was that elevating mtH_2_O_2_ levels could be sufficient to induce breast cancer cell migration, which was tested using a mtDAAO-HyPer mitochondria-targeted system [29] (Fig. 3a). In the presence of *D*-alanine, DAAO increased mtH_2_O_2_ levels in MCF7 and MDA-MB-231 cells (Fig. 3b), which promoted their migration (Fig. 3c). Combining *D*-alanine supplementation and subclinical doses of radiation further increased mtH_2_O_2_ levels (Fig. 3b), but not cancer cell migration (Fig. 3c). We then tested if additional mtH_2_O_2_ generated by mtDAAO-HyPer exacerbated promigratory cell response by inducing cell death pathways; however both cell lines exhibited no change in cytochrome c release, caspase cleavage, or cellular apoptosis/necrosis, as measured by Annexin V/PI staining (Figure S3).

### Transcription factors AP1 and NF-κB participate in breast cancer cell migration induced by subclinical doses of radiation

**Fig. 4:**
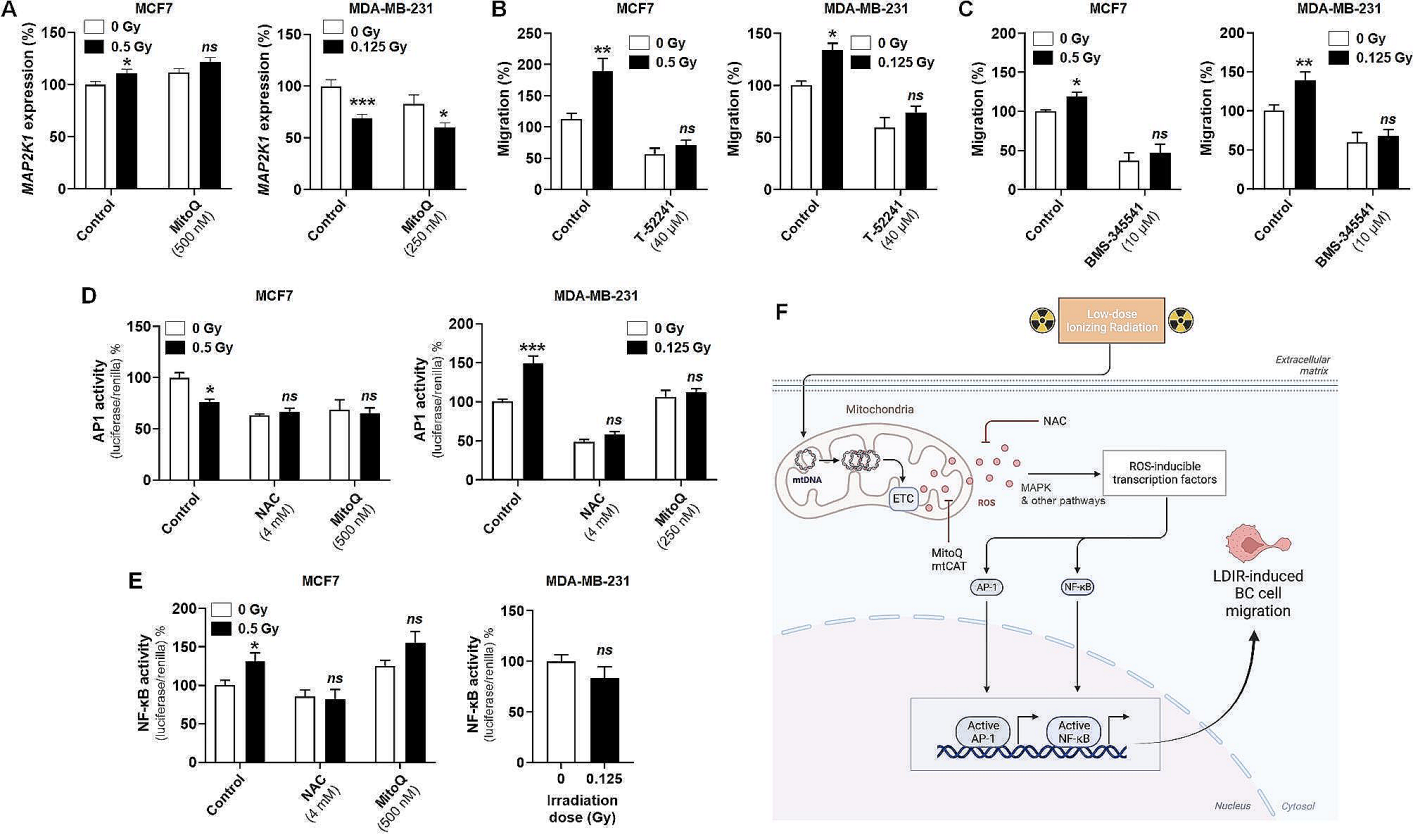
Subclinical doses of radiation activate AP1 and NF-κB in human breast cancer cells. (**A-E**) MCF7 and MDA-MB-231 were irradiated or not with a single dose of 0.5 Gy and 0.125 Gy, respectively, and left to recover for 24 h. (**A**) *MAP2K1/MEK1* mRNA expression was measured in MCF7 and MDA-MB-231 cells 24 h after treatment ± MitoQ (500 nM for MCF7 and 250 nM for MDA-MB-231 cells) (*n* = 9). (**B**) The migratory activity of MCF7 (left graph, *n* = 5–6) or MDA-MB-231 (right graph, *n* = 6) cells was assessed 24 h after treatment ± AP1 inhibitor T-52241 (40 µM). (C) Same as in (B) but ± NF-κB inhibitor BMS-345541 (10 µM) to treat MCF7 (left graph, *n* = 6) and MDA-MB-231 (right graph, *n* = 9) cells. (**D**) Cells were treated ± *N*-acetyl-*L*-cysteine (NAC, 4 mM) or MitoQ (500 nM for MCF7 and 250 nM for MDA-MB-231 cells). AP1 transcriptional activity determined 24 h later using a dual luciferase reporter assay is shown on the left graph for MCF7 (*n* = 6) and on the right graph for MDA-MB-231 (*n* = 6) cells. (**E**) As in (D) but measuring NF-κB transcriptional activity in MCF7 (left graph, *n* = 6) and MDA-MB-231 (right graph, *n* = 6) cells. (**G**) Schematic produced using BioRender depicting the molecular mechanisms supporting the human breast cancer cell migration induced by subclinical doses of radiation. Sequentially, irradiation disturbs the electron transport chain (ETC), promotes mitochondrial ROS (mtROS) production, and activates transcription factors AP1 and NF-κB that trigger breast cancer cell migration. All data are shown as means ± SEM. * *P* < 0.05, ** *P* < 0.01, *** *P* < 0.005, *ns P* > 0.05 compared to control unirradiated cells, by Student’s *t* test

The mitogen-activated kinase (MAPK) pathway has been suggested to promote cancer cell migration in a ROS-sensitive manner [33]. Accordingly, *MAP2K1/MEK1* expression was induced 48 h after a 0.5 Gy dose delivery to MCF7 cells (Fig. 4a). This response was inhibited by MitoQ, linking irradiation-induced mtROS production to MAPK signaling in these cells. However, subclinical dose delivery to MDA-231 cells comparatively repressed *MAP2K1/MEK1* expression independently of the presence of MitoQ (Fig. 4a), indicating that mtROS signaling is multifactorial. We therefore decided to focus on ROS-sensitive transcription factors.

Downstream of the MAPK pathway and of several other ROS-sensitive pathways [34], transcription factors AP1 and NF-κB are known to be ROS-inducible [35–37] and to promote cancer cell migration [36, 38], but whether they could be activated by mtROS 48 h after subclinical dose irradiation was unknown. Inhibiting AP1 with T-55241 reduced the basal migration and blunted the subclinical radiation-induced gain in migration of both MCF7 and MDA-MB-231 cells (Fig. 4c). Similarly, inhibiting the transcriptional activity of NF-κB with IKK inhibitor BMS-345541 blocked basal migration and radiation-induced migration of the two cell lines (Fig. 4b), indicating that both AP1 and NF-κB and participate in the irradiation-induced promigratory phenotype in breast cancer cells. Their relative contribution was further evaluated in our cell models using fluorescent reporters of their transcriptional activities. In MCF7 cells, a 0.5 Gy irradiation reduced AP1 activity (Fig. 4d) but increased NF-κB activity (Fig. 4e), and the two answers were blocked by MitoQ. Comparatively, a 0.125 Gy irradiation activated AP1 (Fig. 4d) but did not modify NF-κB activity (Fig. 4e) in MDA-MB-231 cells. AP1 activation did not occur in the presence of MitoQ (Fig. 4d). Irradiation at subclinical doses can thus activate mtROS-sensitive promigratory transcription factors, but their nature differed across different human breast cancer cell lines (Fig. 4f).

### Targeting mtROS does not reduce the cell killing therapeutic activity of ionizing radiation

We finally aimed to provide some relevance to our observations with respect to photon radiotherapy, generally delivered in 5 fractions per week in clinical settings. One and two fractions of 0.5 Gy induced MCF7 cell migration, but this gain was lost with additional fractions (Figure S4a). Comparatively, MDA-MB-231 cell migration was increasingly induced, reaching a maximum of ~ 4-fold from 2 to 5 fractions.

While subclinical doses of radiation were used throughout this study to model dose deposition at and beyond the tumor margin, most breast cancer cells in clinical settings receive 1.8 Gy to 2 Gy [12]. MitoQ did not interfere with irradiation-induced cell killing at 2 Gy (Figure S4b), supporting its potential use as an adjuvant treatment with photon radiotherapy to counter breast cancer cell migration induced by subclinical doses of irradiation.

## Discussion

In this study, we tested whether long-lasting mitochondrial alterations could promote human breast cancer cell migration. To avoid focusing on idiosyncrasies, we intentionally used two very different human breast cancer cell lines representing luminal A and triple-negative subtypes. They further represent contrasting metabolic archetypes, as MCF7 cells are oxidative whereas MDA-MB-cells are glycolytic in vitro [39]. We identified a sequence of events accounting for migration induced by subclinical doses of radiation (< 2 Gy), commencing with the induction of mitochondrial dysfunction, mtROS production and subsequent activation of redox-sensitive transcription factors. mtROS generation was a shared response between both cell lines. It was still detected 48 h after irradiation and was, thereby, lending itself to pharmacological or genetic repression after irradiation-induced migration.

Our results show that oxidative MCF7 cells demanded a higher irradiation dose to optimally trigger migration than glycolytic MDA-MB-231. This phenomenon can be explained by both increased mitochondrial fitness and lower basal mtROS [40]. Nevertheless, the correlation that we observed between the irradiation dose needed to trigger optimal migration and changes in mtOCR and increased mtROS levels was striking, even if the nature of the mitochondrial dysfunction differed. In the case of MCF7, we postulate that increased undamaged mtDNA content 48 h after irradiation could be the result of an increased mitochondrial turnover and, therefore, mitochondrial abundance. This would logically lead to increased mtROS production via increased cell respiration, which is known to be intrinsically coupled with electron leak from the ETC [41]. Of note, the resulting acquisition of a migratory phenotype depends on (mt)ROS, as shown by the inhibitory effects of NAC and MitoQ, but is also likely modulated by repressors and/or damage to the migratory machinery at irradiation doses > 0.5 Gy [42]. This would explain why increased mtROS production was not always sufficient to trigger MCF7 cell migration. In contrast to MCF7, glycolytic MDA-MB-231 cells displayed an increase in persistent mtDNA damage 48 h after a 0.125 Gy irradiation, associated with a drop in mtOCR despite increased mtDNA content. Here, we suggest a compensatory response to increase mitochondrial biogenesis accompanied by a delay in the clearance of damaged mitochondria. Preserved mtOCR at irradiation doses higher that 0.125 Gy may be explained by the activation of cellular antioxidant defenses above a low dose threshold, as previously proposed by others [43]. This is supported by our observation that an increase in mtH_2_O_2_ by mtDAAO-HyPer did not further induce migration in either cell line, nor did it induce an increase in cytochrome c release or apoptosis/necrosis (Figure S3), which most likely implicates that continual sustained mtH_2_O_2_ generation was not enough to overwhelm cellular antioxidant defense. In the case of MDA-MB-231 cells, increased mtROS production can be directly attributed to mitochondrial defects known to be associated with increased electron leak upon bottlenecking ETC damage [22]. This could then lead to reverse ETC flux associated with Complex I electron leakage [44]. The mitochondrial response of breast cancer cells to subclinical doses of radiation is summarized in Fig. 4g. Of note, although we posit mtDNA alterations as an initial trigger to increase mtROS levels, an additional contribution of mitochondrial content [45], swelling *versus* shrinkage [46] and fission *versus* fusion dynamics [47] is possible.

When electrons leak from the ETC, mitochondrial superoxide is formed followed by mitochondrial H_2_O_2_ generation. With a longer half-life, H_2_O_2_ can permeate the mitochondrial membrane [41] and act as a redox signal to activate ROS-sensitive promigratory pathways [48]. c-Src kinase belongs to one of these pathways: its oxidation activates the TGFβ pathway [22, 49] resulting in the upregulation of the focal adhesion kinase Pyk2 that remodels the cytoskeleton for migration. In breast cancer cells, we further report that subclinical doses of irradiation activate redox-sensitive transcription factors AP1 and NF-κB that cooperate to induce migration. The process would logically depend on the upstream activation of mtROS sensitive pathways, including but not limited to the MAPK pathway [34]. Others reported AP1 activation in RAW 264.7 macrophages [50] and NF-κB activation in lymphoblastoid 244B cells [35] following subclinical radiation doses, indicating that the two transcription factors participate in the general cellular response to such insult. Upon activation, AP1 and NF-κB promote cancer cell migration though inducing the expression of many genes related to cell adhesion, cytoskeleton remodeling, matrix deposition and extracellular proteolysis [51, 52]. Interestingly, all mtROS [23], AP1 [53] and NF-kB [54] are positive EMT regulators in breast cancer cells, offering a likely molecular pathway to explain irradiation-induced EMT [17, 18].

While the single delivery of a subclinical dose of radiation stimulated breast cancer cell migration, it did not trigger in vitro invasion, another necessary phenotype supporting metastasis. Yet, we previously showed that sustained mtROS production is a fundamental and essential characteristic of metastatic progenitor cells in human breast cancer models in mice [55]. It is therefore possible that repeated subclinical dose delivery in fractionated radiotherapy regimen would eventually promote metastasis, which has been suggested by others based on clinical evidence [14]. Exploring this possibility experimentally is a major perspective of our work. If verified, we believe that targeting mtROS could be a preferential therapeutic answer instead of targeting the numerous mitochondrial phenotypes capable of enhancing mtROS production and the multitude of redox-sensitive promigratory pathways downstream of mtROS. Among other drugs, MitoQ selectively inhibiting mtROS formation is a promising candidate as it already successfully passed phase I clinical trials with limited toxicity [56]. For therapeutic mtROS inhibition, noninvasive mtROS imaging in tumors in vivo would also be useful. Specific probes developed for electron paramagnetic resonance bear this promise [57].

Conclusively, our study shows that breast cancer cell migration can be induced by a single subcytotoxic dose of photon irradiation, which can be prevented by mtROS inhibition.

### Electronic supplementary material

Below is the link to the electronic supplementary material.

Supplementary Material 1

## Data Availability

No datasets were generated or analysed during the current study.

## Abbreviations

AP1: Activating protein 1
CAT: Catalase
DAAO: *D*-amino acid oxidase
DAPI: 4’,6-diamidino-2-phenylindole dihydrochloride
DHE: Dihydroethidium
ETC: Electron transport chain
IKK: IκB kinase
MAP2K1: Dual specificity mitogen-activated protein kinase kinase 1 (also known as MEK1)
MAPK: Mitogen-activated protein kinase
MitoPY1: Mitochondria peroxy yellow 1
mtCAT: Mitochondria-targeted catalase
mtDNA: Mitochondrial DNA
mtOCR: Mitochondrial oxygen consumption rate
mtROS: Mitochondrial reactive oxygen species
NAC: *N*-acetyl-*L*-cysteine
NF-κB: Nuclear factor-κB
OCR: Oxygen consumption rate
ORF: Open reading frame
ROS: Reactive oxygen species
SOD2: Superoxide dismutase 2
TGFβ: Transforming growth factor β
