# Supplementary material for: Subclinical dose irradiation triggers human breast cancer migration via mitochondrial reactive oxygen species
Source: Cancer Metab. 2024 Jul 8;12:20. doi: 10.1186/s40170-024-00347-1 (PMC11229245; doi:10.1186/s40170-024-00347-1)
Supplement: Supplementary file 1 — Supplementary Material 1 [file 40170_2024_347_MOESM1_ESM.pdf]

Subclinical dose irradiation triggers human breast cancer migration via mitochondrial reactive oxygen species

Justin Rondeau, Justine A. Van de Velde, Yasmine Bouidida, Pierre Sonveaux

Supplementary Material

Supplementary Methods

Creation of a plasmid encoding a mitochondria-targeted human catalase

To create a mitochondrial-targeted version of the human catalase (mtCAT) open reading frame (ORF), a forward primer (5'-TAA-GCA-GGT-ACC-ATG-TCC-GTC-CTG-ACG-CCG-CTG-CTG-CTG-CGG-GGC-TTG-ACA-GGC-TCG-GCC-CGG-CGG-CTC-CCA-GTG-CCG-CGC-GCC-AAG-GCT-GAC-AGC-CGG-GAT-CCC-GCC-3') encoding the COX8A mitochondrial targeting sequence (MTS) and a reverse primer (5'-TGC-TTA-TCT-AGA-TTA-GGC-GTA-GTC-AGG-CAC-GTC-GTA-AGG-ATA-AGA-GCC-TCC-ACC-CCC-CTC-CCT-TGC-CGC-CAA-GTG-AGA-3') encoding a C-terminal human influenza hemagglutinin (HA) tag in place of the peroxisomal localization sequence (PLS) were used to amplify the ORF of a native catalase plasmid (SinoBio, catalog #HG12084-CY). Using the aforementioned primers, 1 µl of combined forward and reverse primer were mixed with 1 µl of catalase plasmid and 45 µl Platinum™ PCR SuperMix High Fidelity (catalog #12532016) in a 96-well Bio-Rad S1000 Thermal Cycler (Bio-Rad, California, USA) at the following settings: initial denaturation for 2 minutes at 94˚C; 35 cycles of: denaturation for 30 seconds at 94˚C, annealing for 30 seconds starting at 55˚C and increasing every cycle by 1˚C, extension for 1 minute and 30 seconds at 68˚C; 4˚C after cycling until use. Restriction enzymes KpnI (Thermo Fisher Scientific, catalog #FD0524) and XbaI (Thermo Fisher Scientific, catalog #FD0684) were then used according to manufacturer's protocol to create sticky ends for ligation. At the same time, KpnI and XbaI were used to remove the native catalase ORF from its pCMV3 backbone. The digestion reaction was run on a 0.5% agarose gel, dissected from the gel at the correct molecular weight with a clean scalpel,

and the resulting backbone was gel digested and purified using the GeneJET Gel Extraction Kit (Thermo Fisher Scientific, catalog #FERK0692). pCMV3-C-HA backbone and mtCAT ORF sequences were ligated by T4 DNA ligase (Thermo Fisher Scientific, catalog #EL0011) at room temperature for 6 h. The resulting pCMV3-mtCAT-HA plasmid was transformed into One Shot™ TOP10 Chemically Competent *E. coli* (Thermo Fisher Scientific, catalog #C404003), swiped onto agar plates (InvivoGen, catalog #fas-s), and propagated in lysogeny broth (LB) medium. Plasmid DNA was extracted with a PureYield plasmid miniprep system (Promega, catalog #A1222) and quantified by Nanodrop (Thermo Fisher Scientific) according to manufacturer's instructions.

Western blotting was performed as previously shown [1]. Primary antibodies were a rabbit monoclonal anti-catalase (Cell Signaling Technologies, catalog #12980T), and mouse monoclonals anti-HA (Sigma-Aldrich, catalog #H9658) and anti-β-actin (Sigma-Aldrich, catalog #A5441). Secondary antibodies were horseradish peroxidase-conjugated goat anti-mouse (The Jackson Laboratory, catalogue #115-035-003) and goat anti-rabbit (The Jackson Laboratory, catalog #111-035-003).

Staining was also performed as previously shown [1]. Primary antibodies were mouse monoclonal anti-HA (Sigma-Aldrich, catalog #H9658) and anti-ABCD3 (Sigma-Aldrich, catalog #AMAb90995) and a rabbit polyclonal anti-TOM20 (Thermo Fisher Scientific, catalog #PA5-52843) and anti-ABCD3 (Sigma-Aldrich, catalog #HPA032027). Secondary antibodies were Alexa Fluor 488-conjugated goat anti-mouse (ThermoFisher, catalog #A-28175) and Alexa Fluor 488-conjugated goat anti-rabbit (ThermoFisher, catalog #A-11034). Nuclei were stained with 4',6-diamidino-2-phenylindole dihydrochloride (DAPI, 1 µg/mL). All images were acquired with a Zeiss LSM800 confocal microscope, and pictures were compiled using the Zeiss Zen 3.5 Blue Edition software.

Apoptosis assays

Cytochrome c release was detected using a kit from Abcam (catalog #ab65311) according to manufacturer's recommendations. Cytochrome c and caspases were detected using western blotting, following a previously described protocol [1]. Primary antibodies were mouse monoclonals against Cytochrome c (Abcam, catalog #ab65311) and β-actin (Sigma-Aldrich, catalog #A5441) and rabbit monoclonals against caspase 9 (Cell Signaling Technologies, catalog #7237S) and caspase 7 (Cell

Signaling Technologies, catalog #8438S). Secondary antibodies were a horseradish peroxidase-conjugated goat anti-mouse (The Jackson Laboratory, catalog #115-035-003) and a horseradish peroxidase-conjugated goat anti-rabbit (The Jackson Laboratory, catalog #111-035-003). β-actin served as a loading control. Apoptosis and necrosis were detected on adherent cells in 6-well plates using the Annexin V apoptosis detection kit FITC (ThermoFisher, catalog #88-8005-74) according to manufacturer's recommendations. Profiles were determined by flow cytometry on a Canto II flow cytometer (BD Biosciences). A minimum of 10,000 events were acquired for each sample.

Supplementary Results

To engineer a mitochondria-targeted version of human catalase (mtCAT), PCR amplification was performed on a commercially available pCMV3 plasmid containing the human catalase ORF sequence, using primers to add the COX8A mitochondria-targeting sequence (MTS) to the 5' end and to replace the peroxisomal localization sequence (PLS) by a HA tag at the 3' end of the catalase ORF sequence. This insert was ligated in a pCMV3-C-HA plasmid backbone to produce the pCMV3-mtCAT-HA vector depicted in Fig. S2A. This vector thus encoded a mitochondria-targeted HA-tagged human catalase enzyme (Fig. S2B). Effective mtCAT expression was verified by western blotting on MCF7 cell lysate collected 48 h after transfection using antibodies against catalase and against HA (Fig. S2C). We also verified that a 0.5 Gy irradiation did not change mtCAT expression. Its mitochondrial localization was ascertained using immunocytochemistry, showing that mtCAT (detected with an anti-HA antibody) colocalized with mitochondria marker TOM20, whereas native catalase (detected with an anti-catalase antibody) colocalized with peroxisome marker ABCD3 (Fig. S2D). mtCAT and ABCD3 signals were mutually exclusive. Transfecting pCMV3-mtCAT-HA thus allowed mitochondrial expression of catalase in cancer cells.

Supplementary Figures

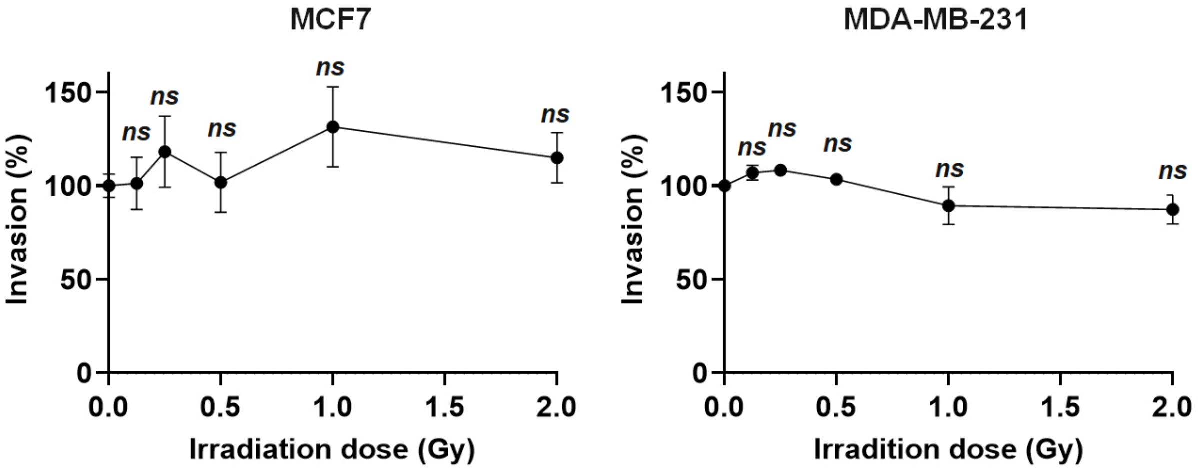

Fig. S1. A single dose subclinical irradiation does not stimulate human breast cancer cell invasion. Cancer cells were irradiated with increasing doses of photons. Their invasion capacities were assayed in transwells for a duration of 24 h starting 24 h after irradiation, with FBS as chemoattractant. MCF7 migration is shown on the left graph (*n* = 4) and MDA-MB-231 on the right graph (*n* = 6). All data are shown as means ± SEM. *ns P* > 0.05; by one-way ANOVA with Dunnett post-hoc test.

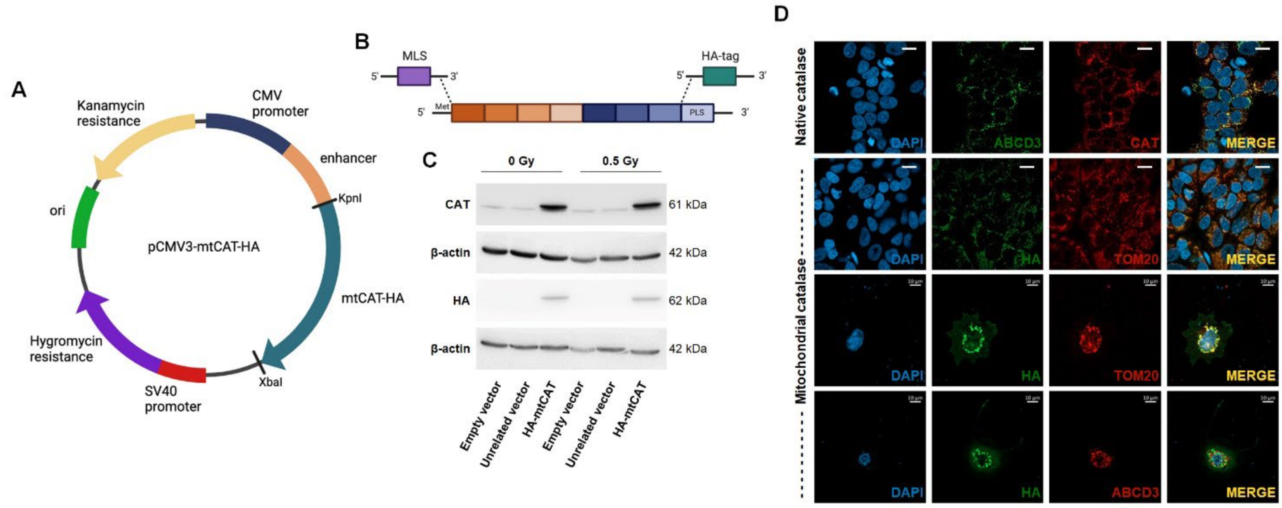

Fig. S2. Generation and validation of a mitochondria-targeted human catalase. (**A**) Schematic produced using BioRender depicting the map of the plasmid construct used to express human catalase (mtCAT). (**B**) Schematic produced using BioRender depicting the mtCAT protein with a 5’ mitochondrial localization sequence (MLS) and a 3’ hemagglutinin (HA) tag (replacing the native 3’ peroxisome localization sequence [PLS]) flanking the human catalase peptide sequence. (**C**) MCF7 cells were irradiated at 0.5 Gy or not, transfected with the plasmid encoding HA-mtCAT or an empty vector, and allowed to recover for 24 h. Protein expression was verified using western blotting with primary antibodies against CAT, HA or β-actin (loading control). (**D**) The subcellular localization of native catalase (top row) in untransfected MCF7 cells and of mitochondrial catalase in mtCAT-transfected MCF7 cells (3 bottom rows) was assayed using immunohistochemistry 48 h after transfection. Primary antibodies were used to detect peroxisome marker ABCD3, mitochondrial marker TOM20, catalase (CAT) or hemagglutinin (HA). Nuclei are stained in blue (DAPI), and merged pictures are provided (bars = 10 μm).

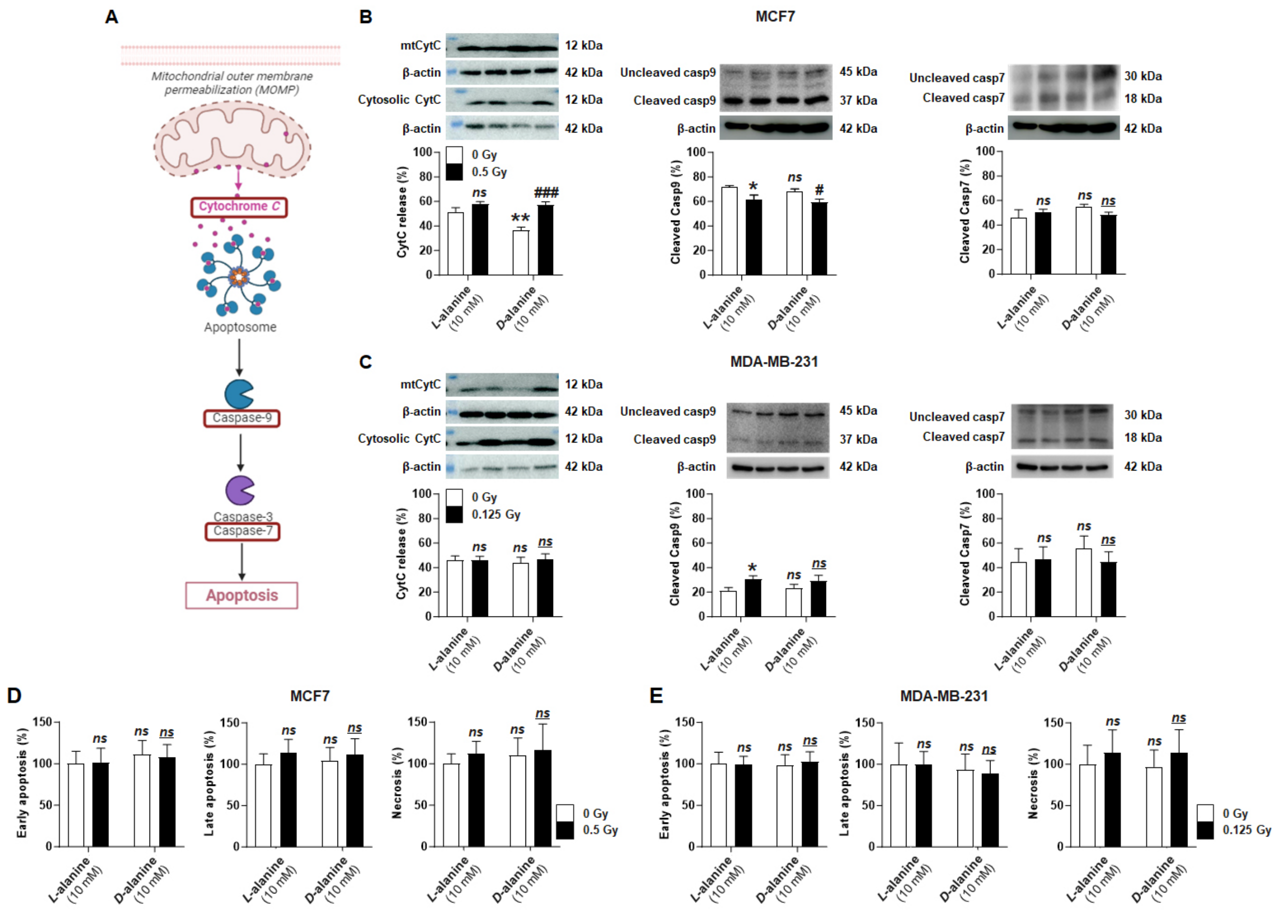

Fig. S3. Excessive mtROS generation causes breast cancer cell apoptosis. (**A**) Schematic produced using BioRender depicting intrinsic apoptosis, where mitochondrial outer membrane permeabilization (MOMP) can sequentially lead to cytochrome c release, and activate/cleave caspases-9 and -7. (**B-E**) MCF7 and MDA-MB-231 cells expressing mtDAAO-HyPer were assayed 48 h after irradiation ± a 24 h of treatment with *L*-alanine or *D*-alanine. (**B**) Cytochrome c release and caspase-9 and -7 activation/cleavage measured in MCF7 cells (*n* = 3-9). (**C**) Same as in (B), but using MDA-MB-231 cells (*n* = 3-9). (**D**) Quantification of early and late apoptosis, and necrosis using Annexin V/PI staining in MCF7 cells (*n* = 5). (**E**) As in (D), but using MDA-MB-231 cells (*n* = 6). All data are shown as means ± SEM. * $P < 0.05$, ** < $P < 0.01$, *ns* $P > 0.05$ compared to nonirradiated cells treated with *L*-alanine; # $P < 0.05$, ### < $P < 0.005$, <u>*ns*</u> $P > 0.05$ compared to nonirradiated cells treated with *D*-alanine, by Student's *t* test (B-E).

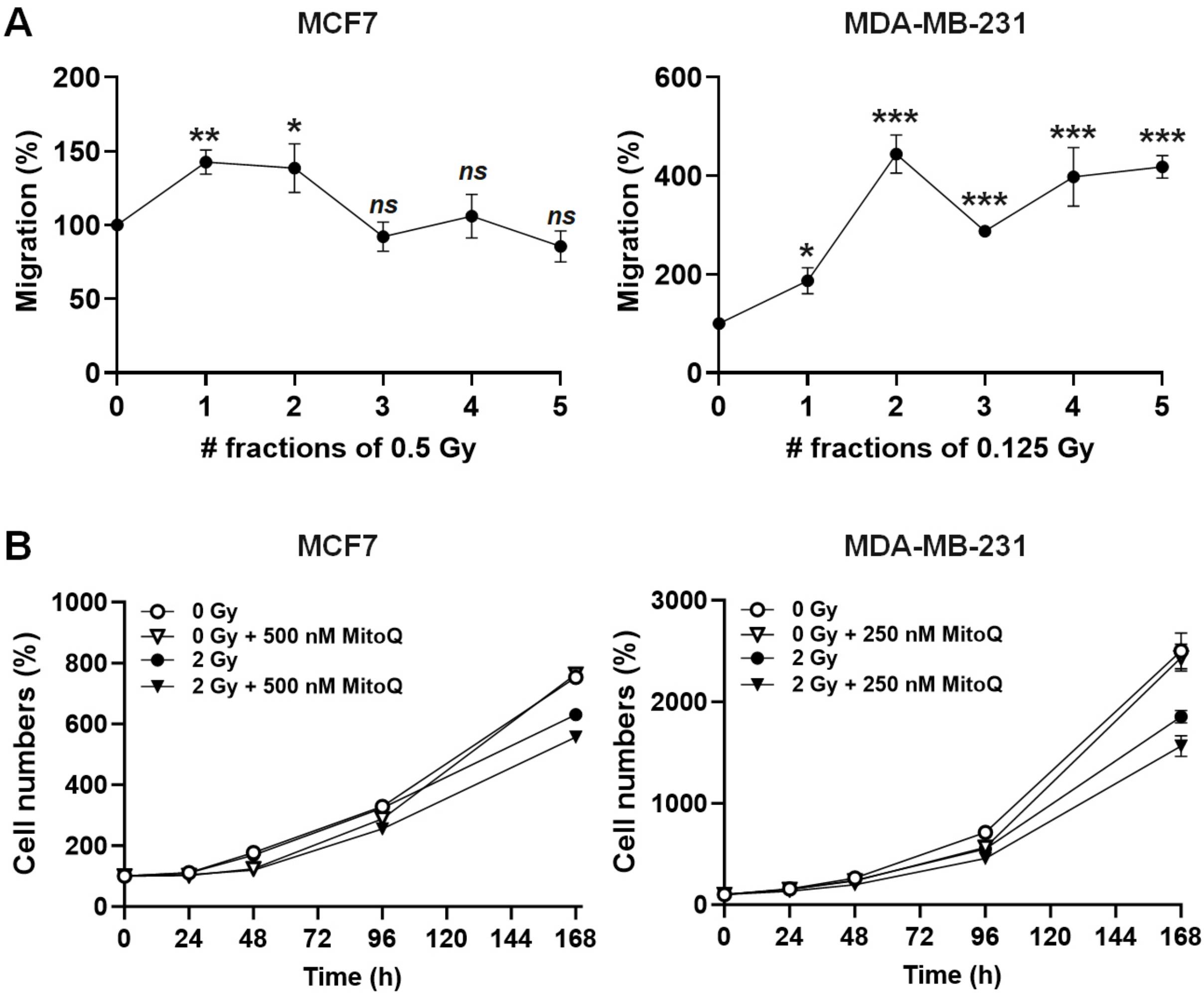

Fig. S4. Selectively targeting mitochondrial superoxide with MitoQ inhibits the migration of human breast cancer cell induced by irradiation at subclinical doses without interfering with irradiation-induced cytotoxicity. (**A**) Cells were irradiated at subclinical doses every 24 h for the indicated numbers of fractions. Their migratory capacities were assayed in transwells with FBS as chemoattractant, starting 24 h after each indicated fraction. MCF7 migration is shown on the left graph (n = 4) and MDA-MB-231 on the right graph (n = 4). (**B**) Cells were pretreated for 24 h with MitoQ (500 nM for MCF7 and or 250 nM for MDA-MB-231), after which they were irradiated at a dose of 2 Gy. The graphs show cell numbers compared to control conditions for MCF7 (left graph, n = 5) and MDA-MB-231 (right graph, n =5) cells. All data are shown as means ± SEM. * $P < 0.05$, ** $P < 0.01$, *** $P < 0.005$, *ns* $P > 0.05$; by one-way ANOVA with Dunnett post-hoc test (A) or by Student's t test (B).

Supplementary Reference

[1] Capeloa T, Krzystyniak J, d’Hose D, Canas Rodriguez A, Payen VL, Zampieri LX, et al. MitoQ inhibits human breast cancer cell migration, invasion and clonogenicity. Cancers (Basel). 2022;14:1516.
